# An Operational Framework for Affect-Adjacent Structure in Plant–Environment Interaction

**DOI:** 10.3390/bioengineering13030287

**Published:** 2026-02-28

**Authors:** Benjamin Calvert, Luc Caspar, Olaf Witkowski

**Affiliations:** 1 Cross Labs, Cross Compass Ltd., Kyoto 600-8558, Japan; 2 School of Life Sciences, University of Essex, Colchester CO4 3SQ, UK

**Keywords:** plant, physiology, affect, echo state network

## Abstract

Plants exhibit complex internal dynamics in response to environmental conditions, yet whether these dynamics reflect structured affective regimes remains unclear. This study investigates whether internal plant signals encode information about affective states defined relationally by sustained environmental conditions. Valence and arousal were operationalised using temperature, humidity, and residual light. Using only internal plant measurements—including bioelectrical activity and volatile gas emissions—we evaluated whether machine learning models could decode affective structure without access to environmental variables. Binary classification revealed that valence was reliably decoded over longer temporal windows, whereas arousal required shorter windows, suggesting distinct underlying timescales. Direct multi-class quadrant classification proved unstable, but an Echo State Network capturing temporal dependencies achieved improved performance. These results indicate that the recorded plants internal dynamics carry a learnable, temporally extended signature of environmentally defined affective regimes, supporting an interpretation of plant affect as embodied environmental engagement.

## 1. Introduction

Plants silently compute the world’s crises (e.g., drought, toxins and climate shifts) through complex processes. Unlike electronic sensors, which provide direct streams of data, plants integrate diverse signals into emergent, nonlinear responses that encode more than the sum of their inputs. Interpreting these responses may allow us to make more holistic decisions about how we act in the world, ultimately guiding choices that benefit both humans and our environment.

The mechanisms by which plants experience and communicate environmental changes through their own umwelt are increasingly well understood. Traits such as phototropism, chemical detection, and bioelectrical signalling represent deeply conserved strategies for sensing and adapting to environmental variability [1]. These processes manifest as measurable bioelectrical responses, respiratory changes, and even physical motions that together form the basis of phytosensing [2,3]. Recent work has demonstrated that plant bioelectrical signals can be used to classify both environmental conditions and external perturbations, suggesting that plant physiological dynamics encode rich, interpretable information beyond simple stress responses [4].

Plants continuously sense and respond to their environment through coupled electrical, physiological, and metabolic processes. Changes in light, temperature, and humidity effects electrical potentials across membranes, stomatal conductance, and respiration-photosynthesis balance, producing measurable signals in both bioelectrical activity and respiratory outputs [5,6,7]. These responses are increasingly understood not as isolated stress reactions but as components of integrated signalling systems that coordinate whole-plant regulation across spatial and temporal scales [8,9].

Beyond their measurable physiology, plants can also be framed as active mediators of environmental information. This work builds on frameworks by Haraway [10], Barad [11] and Braidotti [12], which emphasise relationality, distributed agency, and the decentring of human primacy. Here, the plant is not merely instrumentalised; instead, the sensing system is conceptualised as a hybrid assemblage of plant, human, and machine, in which each participant contributes to environmental cognition.

While research in electrophysiology has demonstrated that plants register and encode environmental perturbations through electrical, hydraulic, and morphological signals [2,3,4,13], such studies have largely remained within the domain of physiological measurement and bio-inspired computation. However, a growing body of scholarship in science, technology and environmental humanities has begun to reconceptualise sensing itself as a distributed and more-than-human process. Gabrys et al. [14] has described how their study *Program Earth* develops infrastructure that embed environmental monitoring into living systems, while Myers [15] experiments with plants as sensing partners to explore entangled modes of perception. Contemporary work in plant cognition has argued that plants exhibit integrative, memory-like, and adaptive signalling dynamics that warrant investigation using frameworks usually reserved for cognitive systems [16].

These perspectives challenge the framing of plants as passive biosensors. By bringing these concepts together, we position our study at the intersection of physiological science and post-humanist theory: the plant is both a reservoir computer encoding environmental dynamics and an active participant in the ecology of sensing. Within the domain of machine learning, increasing attention has been given to feedback-aware and human-informed learning systems. Such work highlights how model evaluation and interpretation can emerge through interaction between computational processes and human judgement [17,18]. The assemblage proposed here extends this spirit beyond human–machine interaction to include biological dynamics as an active component of the interpretive framework. Our system endeavours to place plant physiology, machine inference, and human interpretation as collective participants in sensing and understanding, without reducing any component to a purely instrumental role. In this sense, this work represents an initial exploration of collaborative interpretation across biological and computational substrates.

Importantly, this interpretation does not imply consciousness or subjective experience but emphasises embodied physiological organisation within plant–environment interaction. If plants are understood as embodied, dynamically regulated systems rather than passive sensors, then an operational description of their internal organisation is required; the valence–arousal framework from affective science provides a compact, affect-adjacent language for this purpose without implying subjective experience.

In affective science, affective states are commonly represented within a low-dimensional space defined by *valence* (positive–negative) and *arousal* (low–high activation) [19]. This dimensional framework can be repurposed as an operational description of internal state. Under this interpretation, valence corresponds to the relative favourability of environmental conditions for plant function, while arousal corresponds to the intensity of physiological activation required to respond to those conditions [20]. In this study, we investigate whether plant internal dynamics exhibit affect-like organisation when interacting with sustained environmental conditions.

Affection is often conflated with subjective feeling. However, in affective science these concepts are distinct. Affect can be understood as a structured physiological response to environmental significance, while feeling refers to the conscious, subjective experience of that response [21]. Many organisms, as well as artificial systems, exhibit affect-like regulation without evidence of conscious feeling [22]. In this study, we adopt this physiological definition of affect and explicitly do not claim that plants possess feelings or phenomenological awareness.

In order to accomplish this, we investigate whether changes in a plant’s internal physiological state, specifically bioelectrical activity and respiration-related gas proxies, can be consistently mapped to externally defined environmental conditions framed within an affective space. We focus on a tomato plant (*Solanum lycopersicum*) grown within a controlled chamber in which light, temperature, and humidity are actively manipulated. Internal state is monitored via sensors: electrical signals acquired through electrodes, and metabolic proxies measured as environmental CO_2_ (eCO_2_) and total volatile organic compounds (TVOC). Accordingly, this work is framed as a proof-of-concept study that tests whether a plant’s internal physiological dynamics are sufficiently structured to support affect-adjacent decoding under controlled environmental conditions, rather than as a claim about population-level plant affective organisation.

Rather than relying solely on statistics, we implemented a recurrent computational readout in the form of an Echo State Network (ESN). The ESN was used both to (i) infer the plant’s current affect-like state from environmental inputs and physiological signals and (ii) predict future internal state trajectories under ongoing environmental conditions. This approach treats the plant–environment system as a dynamical process and tests whether a predictive internal representation of plant state emerges.

### 1.1. Plant Electrophysiology and Internal State

Plant cells maintain membrane potentials through ion transport, enabling the generation of electrical signals in response to environmental changes [23]. These include fast action potentials and slower variation potentials, both of which have been observed in response to changes in light, temperature, humidity, and mechanical disturbance [5,6]. These signals occur through vascular and cellular pathways, allowing for coordination of physiological responses [24].

Therefore, bioelectrical activity has been proposed as a candidate indicator of internal plant state, reflecting both immediate stimulus detection and longer-timescale regulatory adjustments [9]. However, electrical signals alone are difficult to interpret without complementary context as similar physiological changes can arise from different drivers [25]. Consequently, changes in processes such as bioelectrical activity is best interpreted not as a direct encoding of specific stimuli but as part of a malleable internal state shaped by environmental history and experience.

### 1.2. Respiration and Volatile Emissions as Metabolic Indicators

Environmental conditions also affect plant respiration and metabolism. Temperature influences respiratory rates, while humidity affects transpiration and stomatal behaviour, thus shaping internal CO2 dynamics [7]. Plants also emit volatile organic compounds (VOCs) whose production can increase under stressors such as elevated light and temperature [26,27]. These signals evolve on slower timescales than electrical responses, complementing bioelectrical measurements.

In this work, eCO_2_ and TVOC were measured using a gas sensor. Within a controlled chamber, they provided reproducible indicators of metabolic and chemical changes associated with environmental transitions.

### 1.3. Valence–Arousal as an Operational Framework

The valence–arousal model provides a common and interpretable means of describing complex affective states [19]. Applied here, valence tells us the ’favourability’ of an environmental condition for tomatoes (e.g., high humidity reducing water stress), while arousal shows the ’degree of physiological activation’ (e.g., elevated temperature increasing stress) [20]. In this study, valence and arousal were defined exclusively by environmental conditions and used as reference labels against which internal physiological dynamics were correlated.

### 1.4. Reservoir Computing as a Framework

Reservoir computing is a computational paradigm in which a dynamical system transforms input signals into a high-dimensional state space. A simple readout function is then trained to map these states onto desired outputs. In this study, an Echo State Network (ESN) was employed as the readout model to infer affective state from plant physiological data. The ESN state update can be described as

(1) $$x\left(t+1\right)=\left(1-\alpha \right)\;x\left(t\right)+\alpha \;f\;\left(W_{in}u\left(t\right)+W\;x\left(t\right)\right)$$

where u(t) is the time-based input, x(t) is the internal reservoir state, $W_{in}$ and *W* are the input and recurrent weights, α the leak rate, and f(·) a non-linear activation function.

In this work, the plant itself is not treated as a reservoir necessarily. Its physiological dynamics constitute a process whose internal states are captured through sensors and projected into an Echo State Network readout for inference [28]. The inputs u(t) therefore correspond to internal plant signals that reflect the plant’s relationship to environmental conditions.

An ESN was chosen based on the potential relationship dynamics of the system and the environment. Firstly, the physiological signals recorded from the plant were continuous data, temporally structured, and exhibited history-dependent responses to environmental changes. ESN’s are designed to retain short-term memory of inputs based on their recurrent dynamics, which allows for sequence decoding and correlation [29]. Secondly, the relationship between the plant and its environment is potentially non-linear. This means that the retention of recurrent weights allowed for linear labelling to be placed on the output and readout layer [30]. Thirdly, ESN’s are robust to noise, which occurs in biological sensing over long time periods [31].

The readout layer was trained to map reservoir states onto discrete affective states in the valence–arousal space, enabling inference of the plant’s current affective state from its internal physiological dynamics:

(2) $$y\left(t\right)=W_{out}\;\left[x\left(t\right);1\right]$$

where y(t) represents predicted conditions (i.e., changes in affective state) that are subsequently mapped onto dimensional affect (i.e., valence or arousal state). Reservoir computing is particularly suited to biological time series data because it requires minimal training of internal dynamics, tolerates noise, and can exploit the natural complexity of physical systems [32,33,34,35]. Plants, as dynamical substrates, have these qualities: high-dimensional responses, non-linear coupling, and environmental sensitivity.

### 1.5. Phytosensing and Discovery

Previous research has demonstrated that plants can act as environmental biosensors [2,4]. Their electrical, thermal, and morphological responses provide high-dimensional encodings of ambient conditions. Beyond electrophysiology, phytosensing encompasses stomatal conductance, volatile organic compound release, and photosynthetic fluorescence [5]. These multi-modal signals encode integrative states that are difficult to reduce to single abiotic variables but which hold potential as ecological readouts of complex conditions.

Motivated by this relational framework, we test whether the internal physiological dynamics of the plant encode interpretable information about sustained environmental regimes. To this end, we employed reservoir computing as a decoding process, testing whether an environmentally defined valence–arousal structure can be inferred from internal plant signals alone. This approach treats plant physiology not as a passive readout of isolated variables but as a dynamical system whose internal states carry learnable structure about ongoing plant–environment interaction.

## 2. Materials and Methods

Our approach built on plant electrophysiology and reservoir computing [2,4] while also engaging with more-than-human sensing frameworks [14,15], situating plants not only as data sources but as active participants in sensing assemblages. Our study employed bioelectrical and respiratory sensors to capture plant state trajectories. The eCO2 and TVOC measurements were treated as proxy indicators of respiratory and volatile emission dynamics within a controlled environment rather than direct measures of photosynthetic rate or metabolic flux, and the sensor was run for 24 h prior to study to attain a baseline value. These were processed through reservoir computing models to infer the affective state of the subject plant and make predictions as to the affective state based on known environmental conditions. This study did not treat this prediction as a passive output: instead, the readout layer was a site of translation between plant computation and human decision-making, embedding post-humanist principles into a practical sensing methodology.

### 2.1. Overview

**Figure 1:**
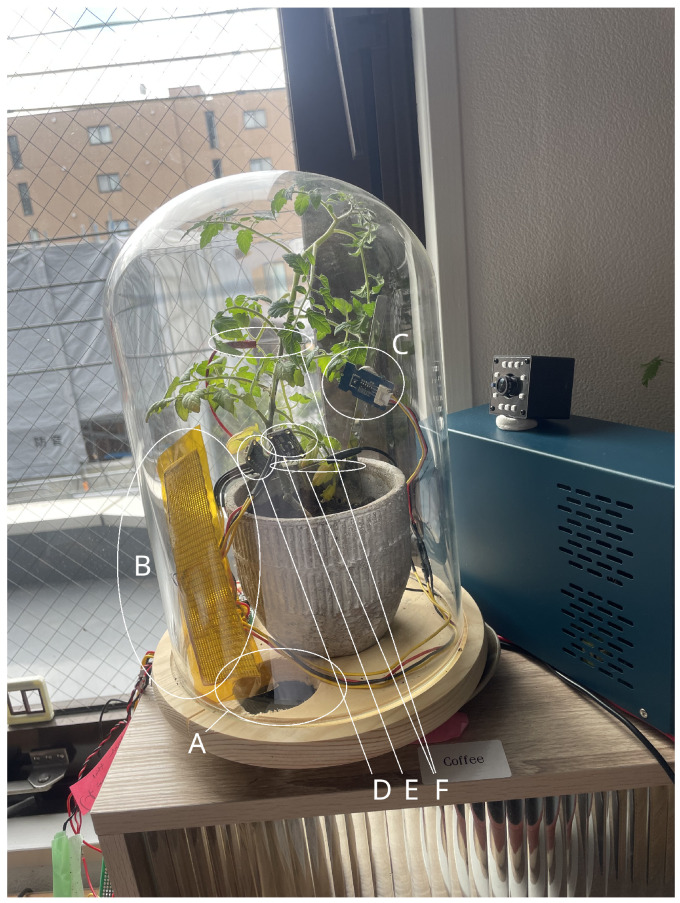
Annotated experimental setup. (**A**) Fan to flux fresh air. (**B**) Heating element providing controlled thermal perturbations. (**C**) SGP30 gas sensor measuring eCO_2_ and total volatile organic compounds (TVOC). (**D**) LTR303 light and infrared sensor positioned at base height. (**E**) HDC302 temperature and humidity sensor located near the plant stem at mid-height. (**F**) Differential electrodes inserted into plant tissue for extracellular bioelectrical recordings (live electrode near canopy, reference electrode basally).

A single tomato plant (*Solanum lycopersicum*) was monitored within a controlled chamber made for environmental sensing and bioelectrical recording. Environmental variables were actively manipulated using a heating pad (Figure 1B) and fans (Figure 1A), while internal plant signals were recorded continuously. All data were logged using a Raspberry Pi 5 (Sony UK Technology Centre, Pencoed, Wales, UK) with environmental and bioelectrical sensors.

### 2.2. Environmental Sensing

Environmental variables were sampled at 0.5 Hz, a rate quick enough to capture chamber dynamics. All sensors communicated via I2C to a Raspberry Pi 5 and are pictured in Figure 1:

- **Light and infrared**: LTR303 sensor (Lite-On Technology; New Taipei, Taiwan) positioned at base height (Figure 1D);
- **Temperature and humidity**: HDC302 sensor (Texas Instruments; Dallas, TX, USA) located near the plant stem at mid-height (Figure 1E);
- **Gas sensors**: SGP30 sensor (Texas Instruments; Dallas, TX, USA) sensor measuring eCO2 and TVOC within the chamber (Figure 1C).

### 2.3. Biosensing

As well as environmental sensors, bio-electricty was measured via differential electrodes. An ADS1115 module was used for bioelectrical signals. Electrodes were placed in the plant tissue itself. The live electrode was placed near the canopy of the plant, with the ground being inserted more basally (see Figure 1F). Signals were sampled at 100 Hz. Although slower than rates used in some electrophysiological studies, this frequency was sufficient to capture electrical fluctuations associated with physiological regulation while maintaining the ability to record over long durations [36]. The inserted probes were used as a detector of extracellular potential changes, as opposed to discrete changes in the action potential of neurons.

The chamber was set near a window in December and January 2025/2026 for circadian rhythm and was subject to ambient temperatures that were optimal for tomato plant growth during the days the office was occupied (approx. 25 °C). The heater was turned on at random intervals to eliminate the potential for rhythmically primed response to temperature increases. The heater was active for between 15 and 45 min, with the length of time also being randomly assigned programmatically. If the temperature of the chamber reached a critical 36 °C, threshold fans were triggered to bring the temperature down while maintaining the temperature above 35 °C to ensure a heat-stressful environment. Fans also activated every 24 h to flux fresh air into the chamber to protect from the over-saturation of VOC’s. Watering occurred once a week, where the chamber lid was removed during a period where the fans and heater were not engaged. The soil was then well saturated with water. No explicit timing or recalibration procedure was applied to the VOC sensor following lid removal; instead, subsequent analyses relied on temporal smoothing and time-series cross-validation to reduce the influence of transient events associated with chamber opening.

### 2.4. Valence and Arousal

In this study, valence and arousal were used as affect-inspired environmental summary coordinates in the 2D space, not as direct measurements of internal phenomenological states. The labels were externally defined from environmental variables and served as targets for evaluating whether internal plant dynamics encode information of affective environmental regimes. Temperature and humidity were used because of their causal relationship in processes such as vapour pressure deficit (VPD). High temperature and low humidity increase transpiration demand as well as stomatal conductance, which usually elicits a fast physiological response. Cooler and more humid environments typically represent more stable physiological states [37,38,39]. Prior studies on tomato demonstrate strong interactive effects of temperature and humidity on growth, endogenous hormone concentrations, and transcriptional responses. This demonstration supports the relevance of these variables for defining distinct physiological regimes [40,41], and the orthogonality of such regimes defines the main dimensions of our state space. Light was included as a positive driver of both axes due to its role in processes such as photosynthesis. However, due to the nature of light in the chamber, whereby light has the effect of increasing temperature and decreasing humidity, the residual effect of light after temperature and humidity’s effect on valence and arousal had been calculated was used.

Environmental variables were first normalised using z-scoring. Residual light was obtained via linear regression of light against temperature and humidity:

(3) $$L=\beta_{0}+\beta_{T}T+\beta_{H}H+\varepsilon$$

where $\beta_{0}$, $\beta_{T}$, and $\beta_{H}$ are regression coefficients. The residual light was then defined as

(4) $$L_{\mathrm{res}}=L-\left(\beta_{0}+\beta_{T}T+\beta_{H}H\right)$$

$L_{\mathrm{res}}$ then represents light that is statistically independent of temperature and humidity within the chamber.

Valence (V) and arousal (A) were then defined as linear combinations of the residual light, temperature, and humidity as follows:

(5) $$\begin{array}{cc} V & =L_{\mathrm{res}}-T+H \end{array}$$

(6) $$\begin{array}{cc} A & =L_{\mathrm{res}}+T-H \end{array}$$

With these equations, valence increases with residual light and humidity and decreases with temperature, while arousal increases with residual light and temperature and decreases with humidity. This structure reflects both the empirical coupling of environmental variables in the chamber and the intended interpretation of valence and arousal as orthogonal affective dimensions defined by environmental factors rather than inferred from internal signals.

Continuous valence and arousal signals were converted into binary affective labels to enable classification-based decoding. Valence and arousal signs were defined as

(7) $$\begin{array}{cc} v(t) & =I[V(t)>0] \end{array}$$

(8) $$\begin{array}{cc} a(t) & =I[A(t)>0], \end{array}$$

where I[·] denotes the indicator function. This yields four affective quadrants corresponding to the Cartesian product of valence and arousal signs:
QuadrantValenceArousal0−−1−+2+−3++

Importantly, these labels were defined by environmental variables and were never inferred from internal plant signals during training. Environmental labels were also aggregated over sliding windows, and the valence and arousal measurements were smoothed using a rolling median. This allowed for a reduction in noise, thus minimising the effect of transient events such as mechanical disturbance (e.g., lid removal), as well as the potential outlying data points this could provide. A classification dead-zone was placed around the zero mark of the state space, reducing the effect of ambiguous state changes that could be caused by chamber operations.

To account for the effect that both circadian rhythm and time-dependent interaction have on the environment by way of routine structure, we performed a null analysis of circular permutations in time shifts. In this control, arousal labels were randomly shifted by both ≥60 min and ≥360 min separately. Valence labels were randomly shifted relative to internal plant signals by offsets of ≥360 min and ≥720 min and training was repeated. Shifts that had a single class fold were discarded.

### 2.5. Reservoir Structure

Temporal decoding was performed using an Echo State Network (ESN) with a fixed random reservoir and a linear readout. The reservoir comprised 300 recurrent units with spectral radius 0.9, leak rate 0.2, and input scaling 0.5. Reservoir weights were randomly initialised using deterministic seeds and remained untrained. A washout of 50 time steps was applied to reduce initial transient effects.

Readout weights were trained using ridge regression with regularisation parameter α=1.0, minimising squared prediction error with an L2 penalty on the readout weights. No gradient-based optimisation or backpropagation-through-time was used and the reservoir dynamics were not trained.

To prevent temporal leakage, the evaluation used forward-chaining time-series cross-validation (i.e., *TimeSeriesSplit*) with five contiguous splits and no shuffling. Feature standardisation was performed within each training fold and applied to the corresponding test fold. For binary decoding of valence and arousal, the linear readout produced a continuous score s=Xw, which was mapped to a probability via a logistic sigmoid and thresholded at 0.5 to obtain class predictions. Quadrant labels were then obtained by combining independent binary predictions for valence and arousal (q=2v+a).

Due to class imbalance, performance was assessed using balanced accuracy and macro-averaged F1 in addition to accuracy. Metrics and confusion matrices were computed exclusively with test segments; early samples were used only for training to preserve causal validity. Testing on early samples would introduce temporal leakage and inflate performance, and, therefore, causal validity was prioritised over full sample coverage. No resampling or synthetic balancing was done, as the aim was to assess decoding under a naturally occurring regime. We additionally evaluated logistic and multinomial readouts; however, these did not improve balanced quadrant decoding, so we retained ridge readouts for consistency with prior reservoir computing practices.

## 3. Results

Data was collected over a two-week period in late December to early January 2025/2026 in an office in Kyoto, Japan. Data was stored in a cloud-based InfluxDB database. This method allowed for off-chip large scale data collection, as well as built-in temporal formatting. IR levels below 1 were filtered out, and while this did not disclude night-time readings, it did eliminate the times when the light sensor failed to write to the database. Using only internal plant signals, we trained an ESN to decode the plant’s location within the defined affective space. Importantly, the model never observed environmental variables directly. Successful decoding implies that internal plant dynamics carry information predictive of the externally defined affective regime and, therefore, of the environment itself, and may support affect-like physiological states without implying subjective or phenomenological experience.

To test the viability at first, linear classifiers were trained to assess valence and arousal independently using internal plant signals only. Input features were gas measurements (TVOC and eCO2) and bioelectrical signals.

Time-series data were segmented into windows, and summary statistics (mean, standard deviation) were computed within each window. After testing, it was decided that binary classifiers would be trained separately for valence and arousal. Balanced accuracy was used as the primary metric to account for class imbalance, as well as F1-score. We chose to limit reported performance statistics to these statistics and confusion matrices to avoid any implication of independent biological replication from window-level metrics. Additional diagnostics (e.g. κ, MCC) were computed during analysis but omitted from the manuscript in the interest of conservative interpretation. Quadrant predictions were then derived by combining the predicted valence and arousal results, rather than being learned directly. This decomposition preserves the separation of affective dimensions.

### 3.1. Environmental Affective Space

**Figure 2:**
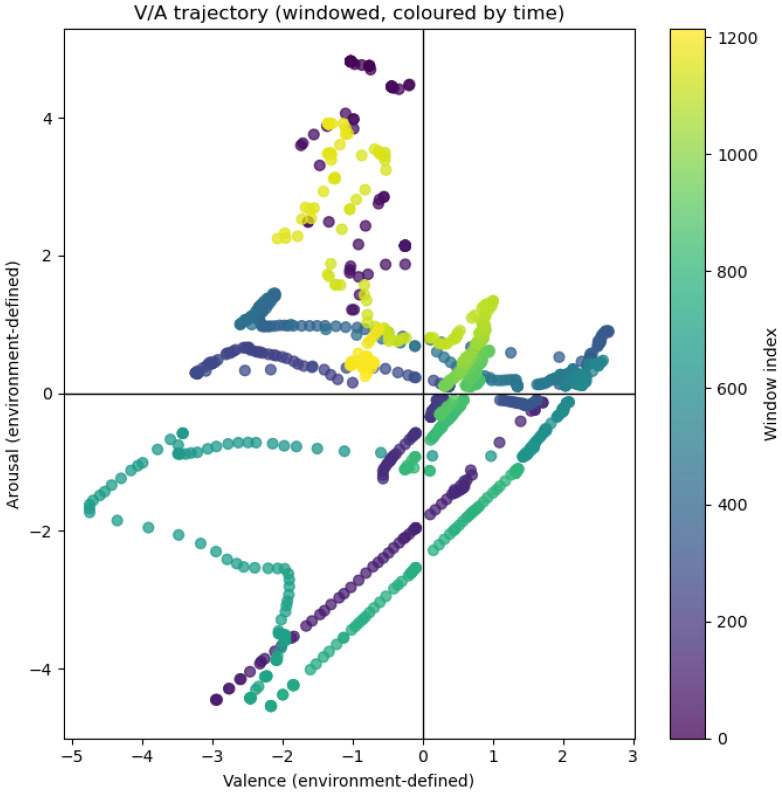
Trajectory of externally defined environmental valence and arousal over a 144 h period. Points correspond to non-overlapping 7 min windows and are coloured by chronological order (window index). Extended residence within quadrants indicates sustained environmental regimes, while smooth transitions reflect gradual changes in environmental conditions.

Across the recording period, environmental conditions occupied all four quadrants of the defined valence–arousal space (Figure 2). Valence and arousal were computed as externally defined summary coordinates from temperature, humidity, and residual light (see Section 2.4).

In Figure 2, each point represents one 7 min aggregated window, and the colour gradient encodes the window index, which increased with time from the start of the experiment. This allowed the temporal progression of environmental conditions to be visualised within the valence–arousal space. The four quadrants correspond to combinations of high/low valence and high/low arousal, defined by the sign of each axis. The trajectory reveals extended residence within specific regions of the state space, with regime durations typically spanning tens of minutes to several hours, with generally smooth transitions between quadrants. This temporal structure indicates that the labels reflect sustained environmental regimes rather than transient perturbations, motivating their use as targets for decoding from internal plant physiological signals.

### 3.2. Classifying Valence from Internal Signals

Using only internal plant signals, binary valence classification exceeded majority-class baselines across multiple temporal window sizes. With a 20 min window and 15 min stride, the classifier achieved a mean accuracy of 0.77±0.19 (n=219,[108,111]), balanced accuracy of 0.82±0.11, and F1-score of 0.803±0.125, compared to a majority baseline of 0.51. The confusion matrix in Table 1 reveals structured errors rather than random misclassification, indicating that internal signals carried information predictive of the environmental valence regime.

### 3.3. Classifying Arousal from Internal Signals

Arousal decoding proved more temporally sensitive than valence. When decoded with windows sizes similar to valence, the decoder provided near-baseline performance. However, windows of 1 min (n=2926,[1103,1823]) provided substantially improved balanced accuracy (0.72±0.16) and F1-score (0.654±0.187) compared to a majority baseline of 0.62, suggesting that arousal-related internal dynamics operate on faster timescales than valence-related dynamics. This difference is consistent with the interpretation of arousal as reflecting rapid physiological responsiveness rather than slow environmental appraisal. As with valence, the confusion matrix found in Table 2 showed structured, adjacent classification errors rather than random misclassifications, concluding that these signals also contain information indicative of environmental arousal regimes.

### 3.4. Control Analysis

To test whether decoding performance could be explained by predictable chamber routines or diurnal structure, we performed null test permutations by circularly shifting time. When arousal labels were misaligned from internal plant signals by ≥60 minor ≥360 min, balanced accuracy collapsed to near-chance levels (0.503±0.054 and 0.508±0.053, respectively). Valence followed a similar trend, collapsing to near-chance levels 0.500±0.054 at ≥360 min and 0.513±0.057 at ≥720 min. This collapse occurred despite identical model training and evaluation. In contrast, the unshifted condition achieved significantly higher performance (arousal balanced accuracy = 0.715±0.16; valence balanced accuracy = 0.816±0.11), indicating that decoding depends on temporally-aligned plant internal dynamics rather than routine-aligned environmental structure.

### 3.5. Quadrant Classification Through Combined Binary Classifier

Direct classification of affective quadrants from internal signals resulted in unstable performance and unstructured confusion matrices. However, breaking down the task into independent valence and arousal classifiers and combining them produced more interpretable results. However, quadrant classification accuracy remained modest (0.51–0.54); balanced accuracy improved relative to chance (0.51–0.53 vs. 0.25 for chance).

Performance varied substantially across cross-validation folds. Some folds lacked full quadrant coverage, inflating the variance and limiting macro-averaged metrics. These limitations provide further reasons for an ESN decoding approach that preserves temporal continuity rather than relying on static windowed summaries.

### 3.6. Echo State Network Classification

ESN decoding substantially increased windowed sample numbers (*N* = 26,630) and stabilised performance across cross-validation folds. Quadrant classification accuracy reached 0.59±0.29, with balanced accuracy of 0.53±0.21, exceeding chance levels (0.25) and outperforming the window-based approaches. Misclassification occurred primarily between adjacent quadrants rather than opposing affective states. To assess whether quadrant decoding performance was limited by the choice of readout, we evaluated logistic binary readouts, with class balancing and threshold tuning, and a direct multinomial readout trained on the same reservoir states. Under identical windowed time-splits, these alternatives produced comparable macro-balanced accuracy and exhibited similar confusion patterns. We therefore kept ridge regression readouts for the main analysis for consistency with standard reservoir computing practices.

## 4. Discussion

This study explores the hypothesis that plants occupy affective states relative to their environmental conditions and that these states are reflected in internal physiological dynamics that can be learned by machines. Importantly, given the limited population size and novelty of the work presented, the findings discussed hereafter are intended as a proof of concept. This study suggests that internal physiological dynamics are sufficiently structured to permit informed decoding, with the hope of motivating further investigation with biological replication. Affective state was defined not as a subjective phenomenological property but as a relational construct arising from sustained environmental regimes. The internal signals of the plant were treated as an embodied record of these regimes, and machine learning was used to determine whether affective structure could be decoded without direct access to environmental variables. We follow the post-humanist perspective that affect is not an inherently conscious notion and that detection is not only conducted by machines; rather, humans, machines, and biological systems can form coherent assemblages that expand human interpretive and experiential access to non-human dynamics.

Across all classification approaches, internal plant signals were found to contain information predictive of externally defined affective-adjacent regimes. However, performance varied substantially depending on both the affective dimension considered and the temporal structure of the model. The reasoning behind the difference in timescales for effective decoding is based on plant biology and the way this is captured.

Binary classification of valence consistently exceeded majority-class baselines and remained relatively stable across window sizes, indicating that valence-related information is distributed over longer temporal scales. Valence is shown in this model to be a representation of the favourability of the current regime. This regime favourability is not an instantaneous reaction to abrupt changes but a slower integration of metabolic, hydraulic, and acclimatised processes over minutes or hours [7,42]. This capturing of valence interestingly correlates with the claims of Lyon and Kuchling [43], whose work claims that valence is a mere mechanism to distinguish between “advantage and harm” and that this function should not be limited to conscious beings.

In contrast, arousal decoding was highly sensitive to window duration, with performance improving markedly at short timescales. Arousal is capturing fast transients in plant physiology, associated with abrupt perturbations in the environment. Analysis from studies such as those by Fromm and Lautner [5] and Lawson and Blatt [44], as well as the classic work on the subject from Volkov [6], highlights the rapid, “almost instantaneous” [44] effect environmental changes can have on the physiology of a plant. The activations of such changes in electrophysiology are highlighted in the resultant arousal measurements. This temporal dissociation is consistent with established valence–arousal models in affective science, in which arousal tracks rapid physiological mobilisation while valence reflects sustained environmental appraisal [45].

Direct multi-class classification of affective quadrants from static windowed features was unreliable, producing unstable performance and random confusion matrices. Decomposing the problem into independent valence and arousal classifiers improved interpretability and revealed structured errors, with misclassification primarily occurring between adjacent quadrants. Adjacent quadrant misclassification is a sign of structural errors rather than random ones. It reveals that when producing errors, the decoder primarily got one quadrant correct while misclassifying the other. Given that the boundaries created are artificial and discrete and data points near those borders will be similar in structure, classification of continuous dynamic data will not be perfect. This does not delegitimise the effect of the decoder but rather highlights the effect of discretising continuous data. This supports the interpretation that affective structure is present in internal signals but not well captured by static, multi-class classification systems.

Echo State Network decoding further improved performance by preserving temporal continuity in internal plant dynamics. Compared to window-based classifiers, the ESN achieved higher average accuracy and reduced fold-to-fold variability, indicating that affective information is distributed across time rather than being localised to individual windows. Similar to binary quadrant classification, the misclassification occurred primarily between adjacent quadrants as opposed to random affective state changes. This indicates that the ESN captured affective structure based on temporal dependencies at a rate much better than chance. However, performance was far from perfect. This reflects the fact that although temporal structure in internal plant dynamics is informative of affective regime, the mapping between internal state and environmental affect is neither deterministic nor fully captured by the present model.

Although not perfect, this configuration presents decoding as part of an interpretive loop that expands human access to non-human temporal organisation. Contemporary machine learning increasingly recognises that model behaviour and interpretation are shaped not solely by algorithmic optimisation but by structured feedback between humans and computational systems. Studies such as those by Xu and Zhang [46], Wong and Tan [17], Glickman and Sharot [47], and Davidovic et al. [48] illustrate how diversified feedback influences model evaluation and alignment. The present ESN architecture aligns conceptually with this collaborative spirit and the broader sensory augmentation movement. In this sense, the reservoir computer functions not merely as a classifier but as an interface between biological temporal dynamics and human-readable structure, situating plant physiology, machine inference, and human interpretation within a shared sensing ecology.

Finally, several limitations should be acknowledged. Firstly, this study focused on a single plant species under controlled indoor conditions, limiting generalisability, although the species chosen was a model species for experimentation in dynamics capturing. Secondly, classification performance varied across folds, reflecting both class imbalance and the inherently probabilistic relationship between internal plant dynamics and environmental regimes. Finally, while Echo State Networks captured temporal structure more effectively than static classifiers, they are simplified models of plant physiology and do not capture the full picture of biochemical and electrical signalling. Future work should explore multi-plant replication, non-linear affective mappings, and richer temporal models to further characterise the structure and limits of plant affective dynamics.

## 5. Conclusions

Taken together, these results indicate that internal plant signals can support structured affective-adjacent regimes correlated with their environmental conditions and that these regimes are learnable by machines based on internal dynamics. Rather than supporting a simplistic stimulus–response model, the findings point toward a temporally based, embodied form of affective organisation in plants that is probabilistic and history-dependent. While the present models are limited, the results are nevertheless consistent with internal plant dynamics occupying an affective-adjacent space that can be inferred and used to characterise environmental conditions.

This study treated affection as a relational and functional construct of the environment. Valence and arousal were operationalised as structured responses to environmental regimes, defined in terms of environmental data and internal physiological states, without reference to mental states. This distinction is aligned with works such as those by Lyon and Kuchling [43] and Dehaene [49], who state that affect is a non-conscious form of environmental engagement. In the context of plants, this view is consistent with arguments that plant behaviour reflects sophisticated adaptive regulation and internal integration without necessitating subjective awareness or consciousness [50]. From this perspective, affect does not need to be conscious in order to be real, structured, or functionally significant. By separating affection from consciousness, this work contributes to a broader effort to understand affective organisation as a property of embodied systems operating across biological scales. This study has shown that internal plant dynamics carry information predictive of the externally defined affective regime and, therefore, of the environment itself. It therefore could form a basis of support for affect-like physiological states without implying subjective or phenomenological experience, just as many physiological and regulatory processes in animals operate affectively without entering awareness.

In this sense, affect in plants need not be understood as emotion but as a temporally extended mode of environmental engagement that can be inferred, but not reduced, through internal state dynamics. The affective space constructed in this study is not exclusive to plants, but has some analogy with dimensions humans themselves inhabit, suggesting the possibility of future human–plant interfaces grounded not in anthropomorphism but in shared affective structure.

## Figures and Tables

**Table 1:** Confusion matrix for binary decoding of valence from internal plant signals.

	Predicted	
True	V^−^	V^+^
V^−^	67	11
V^+^	31	71

**Table 2:** Confusion matrix for binary decoding of arousal from internal plant signals.

	Predicted	
True	A^−^	A^+^
A^−^	656	344
A^+^	546	889

## Data Availability

The GitHub repository for this work can be found at https://anonymous.4open.science/r/plant_va-3301. Repository created 4 February 2026.
